# Temporal evolution of structure property relationship for UV+RH artificially weathered material extrusion additive manufactured PLA

**DOI:** 10.1038/s41598-026-41192-0

**Published:** 2026-03-02

**Authors:** Mirza Faizaan, Satish Shenoy Baloor, Srinivas Nunna, Suhas Yashwant Nayak, Rohit Nandakumar Shenoy, Chandrakant Ramanath Kini, Claudia Creighton

**Affiliations:** 1 Manipal Institute of Technology, Manipal Academy of Higher Education, Manipal, India; 2 Institute for Frontier Materials, Deakin University, Warun Ponds, VIC 3216 Australia; 3 School of Engineering, RMIT University, Melbourne, VIC 3000 Australia

**Keywords:** 3D Printing, Additive manufacturing, FDM, PLA, Temporal evolution, Tensile properties, Artificial weathering, Chemistry, Engineering, Materials science

## Abstract

This study addresses the underreported temporal evolution of weathering on material extrusion additive-manufactured (MEX-AM) polylactic acid (PLA). Overcoming the limitation of arbitrary exposure durations in existing literature, a time-dependent investigation was conducted on MEX-PLA samples subjected to prolonged artificial weathering for up to 2000 h using a UV-B equipped accelerated weathering chamber with controlled relative humidity. The changes in mechanical, chemical and thermal properties were analysed at 200-hour intervals. The results revealed a time-dependent degradation mechanism characterised by β-chain scission. FTIR analysis confirmed the formation of C = C groups and the progressive loss of H groups, indicating substantial material degradation. Furthermore, DSC and XRD data demonstrated a progressive increase in crystallinity with prolonged exposure, leading to a significant reduction in tensile strength. At the same time, the tensile modulus remained relatively stable for MEX-AM PLA.

## Introduction

Assessing the outdoor conditions of polymers to evaluate their degradation in mechanical, chemical and thermal properties has been gaining interest due to the desire for maximising the lifetime of polymer structures. As weathering conditions are very local and complex to replicate due to the nature of non-controlled variables such as atypical hot and cold days, changing wind patterns, etc., accelerated weathering is considered representative of the outdoor conditions^1^. The growing awareness of sustainable bio-based polymers has increased the demand for polylactic acid (PLA) across industries^2^. Owing to its low cost, ease of use, better mechanical properties than other 3D printable plastics and inherent biodegradability, PLA is the most common used thermoplastic in material extrusion additive manufacturing (MEX-AM), widely called Filament Fusion Fabrication (FFF) or Fused Deposition Modelling (FDM). Further, the advent of MEX-AM and rapid prototyping technology has transformed the polymer/plastic accessibility in every household^3^.

Currently, very little literature is focused on the accelerated weathering of PLA and its composites^1^. We observed that predominantly arbitrary exposure durations have been adopted in the literature. Table 1 summarises the tensile and flexural strength degradation caused by ultraviolet (UV) and relative humidity (RH) reported in the literature. The duration of exposure measured irradiance, percentage of decrement in mechanical strength, and testing standards adopted in each study are summarised.

**Table 1:** Summary of PLA artificial weathering caused by UV degradation.

Exposure condition (hours; mW/m^2^)	% Decrement	Testing standard	Remark	References
2000 h; 890	FS – 99%	ASTM G154		^4^
600 h; 39	TS – 77.6%	–		^5^
700 h; 60,000	TS – 43.5%	ISO 4892-2	Irradiance calculation error likely	^6^
500 h; 760	TS – 12.5%	–		^7^
600 h; 39	TS – 74%	–		^8^
200 h; 490	TS – 61.1%	ISO 4892-3		^9^
300 h; 490	FS – 83.2%	ISO 4892-3		^10^
1200 h; 890	TS – 66.15%	ASTM G154		^11^
750 h; 680	TS – 37.5%	ASTM G154		^12^

FS, flexural strength; TS, tensile strength.

Notably, PLA is susceptible to UV light and moisture, such as photo-degradation and hydrolytic degradation^3^. It is noted that PLA absorbs UV below 270 nm, and the degradation rate increases with temperature and RH. PLA polarity favours hydrolysis and photolysis^1^. The intensity of photodegradation is highest for carbon arc lamps, followed by metal halide lamps, xenon lamps and outdoor exposure^13^. Photochemical degradation of PLA is widely studied in the literature^3,14–16^, and the degradation reaction is summarised in Table 2. PLA degrades following Norrish Type I and Norrish Type II reactions, beginning with homolysis of CH_3_ groups (stage 1) and photochemical cleavage of the polymer chain, indicative of Norrish Type I behaviour (stage 2). The reaction progresses to the continued abstraction of H groups (stage 3), confirming Norrish Type II reaction, leaving free radicals (stage 4) that promote further cross-linking and chain scission (stage 5). Gonzalez-Lopez et al.^1^ reviewed the effect of additives, fillers and reinforcements on the photo-degradation and hydrolytic degradation of PLA and its biocomposites, noting that the degradation rate of PLA depends on the initial molecular weight, sample dimensions, polymer crystallinity and filler/reinforcements.

**Table 2:**
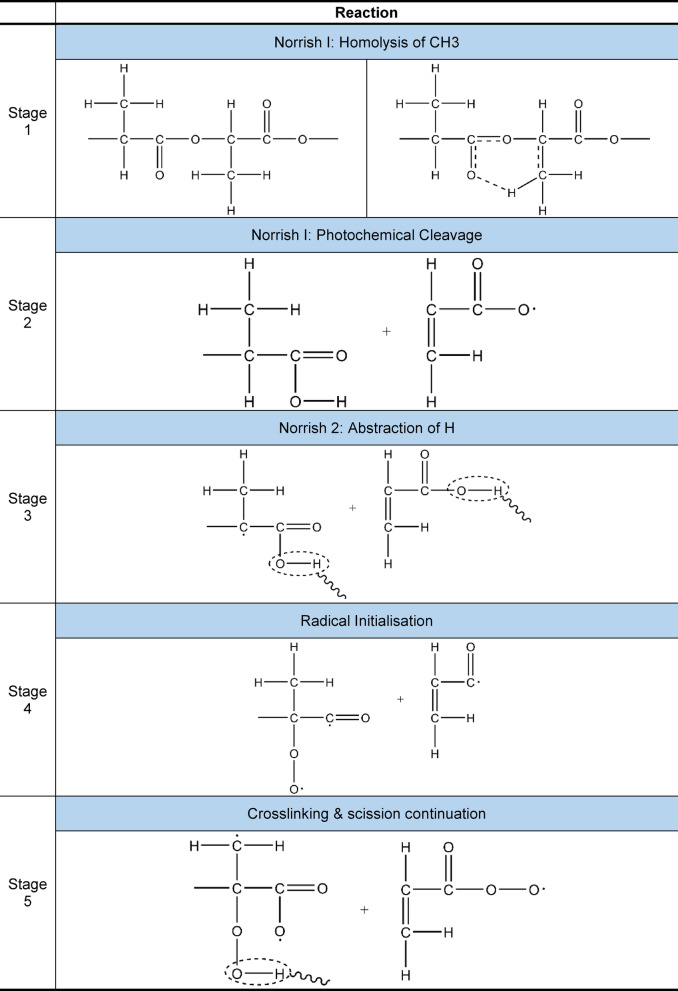
Photo-degradation mechanism of poly(lactic) acid^3^.

With the increasing interest in PLA for MEX-AM and PLA-based composites as a bio-sourced biodegradable and sustainable alternative, it is imperative to understand the effect of weathering and environmental conditioning on the polymer’s mechanical, chemical and thermal properties, especially as a product of MEX-AM. We know from literature that MEX-PLA tends to absorb twice as much water and has a higher crystallinity than injection moulded PLA^17^. The FDM process-induced voids promote water uptake and induce residual stresses and the formation of cracks^18^. Chopra et al. exposed MEX-PLA to outdoor conditions in Aurangabad, India and elucidated the degradation mechanism of PLA^3^. The authors further studied the effect of infill density and pattern on the degradation, confirming that samples printed with less than 100% infill density suffered higher degradation, resulting in the deterioration of their tensile performance. A similar decrease in tensile performance was observed in weathered PLA processed using methods other than FDM^1^. Changes in thermal properties and crystallinity of artificially weathered PLA note that accelerated weathering forces rearrangement of amorphous PLA chains to highly ordered spherulites, resulting in a significant increase in the crystallinity of the PLA up to 60%^9,19–21^.

The literature on the weathering of PLA focuses primarily on the effect of biocomposites, polymer blends, and fillers as opposed to neat PLA subjected to prolonged exposure times. Also, the literature adopts arbitrary exposure times to evaluate the degradation of these PLA composites^1^. A reliable time-based trend representation is still missing in the literature. Furthermore, limited literature is available on the weathering of MEX-AM-based PLA. Thus, an experiment was designed to evaluate the time-dependent degradation of MEX-PLA specimens.

This study examines the temporal evolution of UV + RH accelerated weathering on MEX-PLA tensile coupons. The samples were subjected to artificial weathering for up to 2000 h to establish the trends in polymer properties and mechanical strength degradation that are currently missing in the literature. Fourier transform Infrared Spectroscopy (FTIR) was carried out on the samples before tensile testing, followed by X-Ray Diffraction (XRD) and Differential Scanning Calorimetry (DSC) on the gauge length of the tensile coupons. The percentage of crystallinity and crystallite size were calculated from the XRD spectra, and the thermal behaviour of weathered MEX-PLA was examined using DSC. Finally, the structure-property relations are discussed.

## Materials and methods

### Fabrication of tensile test coupons

Samples were fabricated on the open-source Creality Ender 3 V2 desktop 3D printer using a commercial 1.75 mm diameter eSun PLA+ filament (white). A 0.6 mm nozzle was used to fabricate 100% concentric infill samples following ASTM 638D Type − 1^22^. A 0.15 mm layer thickness, 20 mm/s print speed at 210 °C with two top/bottom 45° raster layers was selected to minimise voids in the test coupons. Tensile tests were performed on the BISS 50KN UTM with a 5 mm/min strain rate. Three samples were tested every 200 h, up to 2000 h of UV + RH exposure, to ensure repeatability, and tensile performance degradation over prolonged exposure times was analysed. The unexposed samples are referred to as ‘0 hours’ for convenience. Thus, 33 samples were fabricated for accelerated weathering, while 30 test coupons were subjected to extreme conditions.

### Accelerated weathering

**Fig. 1:**
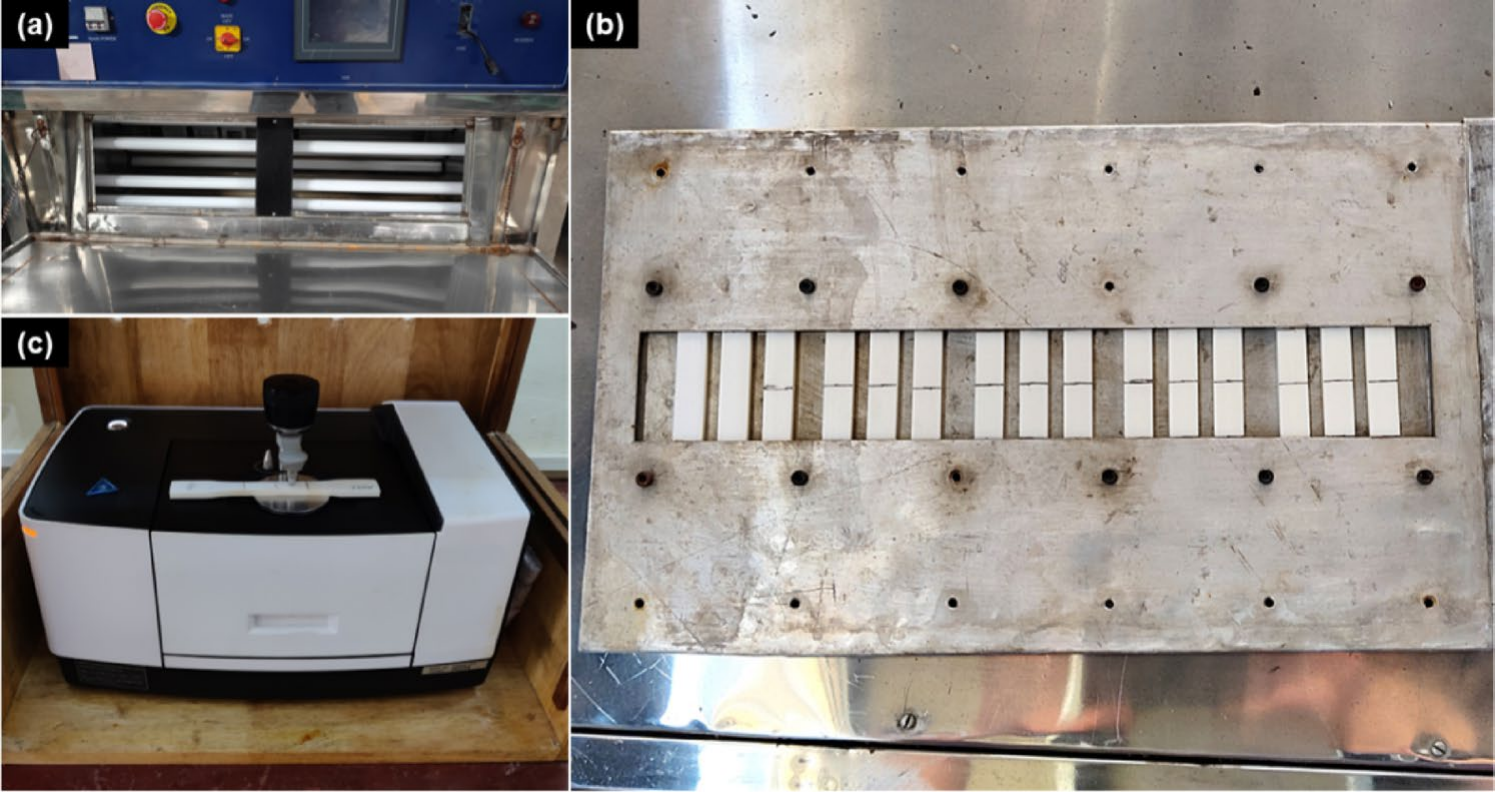
(**a**) UV + RH weathering chamber used for ageing FDM-PLA. (**b**) custom-made tensile sample mount to expose only the 50 mm gauge length of the tensile test coupons. (c) ATR-FTIR probe on the tensile test coupon gauge section exposed to cyclic UV + RH.

The weathering tests were carried out according to ASTM G151 and G154 standards for ultraviolet (UV) weathering of non-metallic materials^23,24^. An accelerated weathering chamber equipped with fluorescent UV-B lamps rated with a peak spectral intensity of 306 nm and a typical irradiance of approximately 0.49 W•m-2 was used for the cyclic 8 h UV and 4 h condensation (relative humidity (RH)) at 60 °C and 50 °C, respectively. Figure 1a shows the weathering equipment, and the patented sheet metal tensile sample holder^25^ designed to expose only the gauge length of the test coupons while preventing warping of the samples under prolonged temperature exposure is given in Fig. 1b. The samples were flipped every 7 days to expose both sides of the tensile test coupons.

### Fourier transform infrared spectroscopy (FTIR)

To assess the surface degradation of 3D printed PLA, Fourier Transform Infrared (FTIR) spectroscopy was employed. A diamond Attenuated Total Reflectance (ATR) probe was used on the exposed surface of the gauge length of the tensile coupons. FTIR spectra were collected every 200 h of cyclic exposure, with 50 scans recorded per sample on a Shimadzu IRSpirit spectrophotometer (Fig. 1c). A total of 2526 data points were recorded for each FTIR measurement. FTIR measurements were conducted immediately after each weathering interval and prior to tensile testing, ensuring that the recorded spectra correspond to the surface condition at the time of mechanical evaluation. Subsequently, the recorded spectra were processed on MATLAB, involving baseline correction, normalisation, and smoothing. As is used alternatively in literature, the carbonyl index (CI) was calculated by determining the ratio of both area under the curve and intensity, under the carbonyl (C=O) peak at 1750 cm^− 1^ and methylene (CH_2_) peak at ~ 3000 cm^− 1^, respectively^1,26,27^. The CI is a dimensionless parameter that provides valuable insights into the degree of polymer chain scission and crosslinking, key indicators of material degradation and embrittlement.

### X-ray diffraction (XRD)

The 50 mm gauge length sections were carefully cut using the Struers Accutom-50 precision cutter. XRD measurements were carried out on the X’PERT PRO with a Cu X-Ray tube emitting a wavelength of 1.5406Å for a range of 5–50° with a scan time of 2.5s per step. XRD data collected for every 200 h of exposure were batch processed and plotted on MATLAB, including calculating crystallinity [%] and crystallite size at different peaks. The per cent crystallinity was calculated by integrating the area under crystalline peaks, and the crystallite size (L) was calculated using the Scherrer equation:

$$\:L=\:K\lambda\:/\beta\:cos\theta\:$$

where K = 0.9, λ = 1.5406 Å, β is the full-width half maximum (FWHM), and θ is the scan angle of the respective peaks. The calculated crystallinity values are dependent on baseline selection and peak deconvolution assumptions. Accordingly, crystallinity is reported here as a relative measure to track temporal evolution rather than as an absolute quantitative value.

### Differential scanning calorimetry (DSC)

DSC was performed on a Netzsch DSC 214 Polyma in dynamic mode. The gauge section of the tensile coupon subjected to accelerated weathering conditions was carefully scraped using a scalpel to collect approximately 10 mg of the sample. These were heated from 20 °C to 300 °C with a 10 °C/min heating rate. Only the first heating was recorded, and the changes in glass transition (Tg), cold crystallisation (Tc), and melting (Tm) were analysed for all eleven samples, including unexposed (0 h) up to 2000 h in steps of 200 h of UV + RH exposure.

## Results and discussion

### Fourier transform infrared spectroscopy

**Fig. 2:**
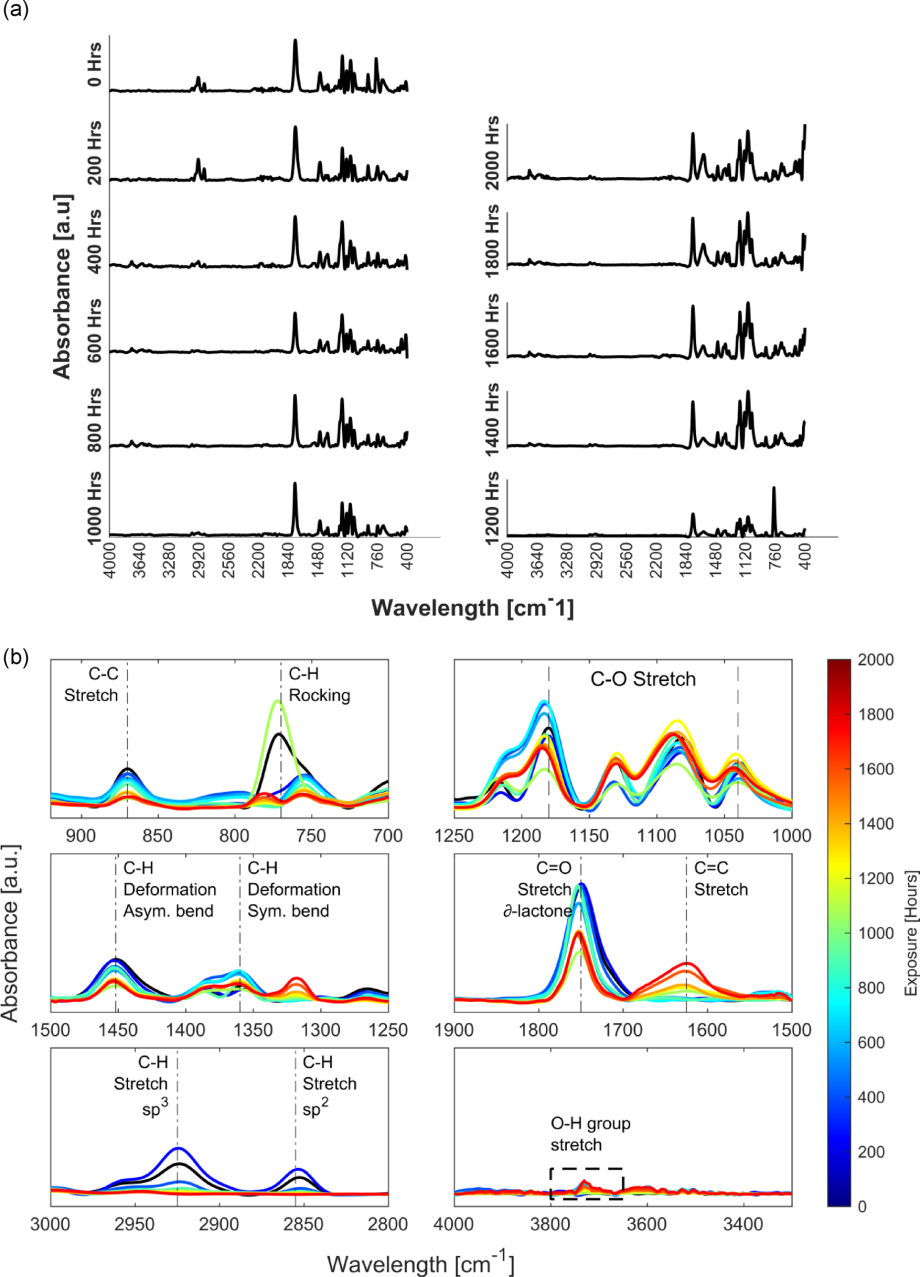
(**a**) FTIR spectra for weathered samples in steps of 200 h of exposure up to 2000 h, and (**b**) illustrating the change in absorbance intensity of normalised spectra over continued exposure.

FTIR spectra were collected using an ATR probe on the gauge length of the tensile coupons exposed to UV + RH weathering in an accelerated weathering chamber. FTIR spectra was captured for every 200 h of weathering, starting from 0 h (unexposed) up to 2000 h of exposure, as seen in Fig. 2a. The collected data were normalised and smoothed using MATLAB, and peaks were identified. The peak intensity, corresponding functional group and its vibrations are tabulated in Table 3. The changes in spectra over continued exposure are illustrated in Fig. 2b.

**Table 3:** Peak assignment for artificially weathered UV + RH FDM-PLA.

Wavelength (cm^−1^)	Bond	Vibration
3800–3600	–O–H group	Stretch
3000–2900	sp3 C–H group	Symmetric stretch
2900–2800	sp2 C–H group	Asymmetric stretch
~ 1750	C=O δ-lactone	Stretch
1700–1600	C= C group	Stretch
~ 1450	C–H deformation	Asymmetric bend
~ 1350	C–H deformation	Symmetric bend
1250–1000	C–O group	Stretch
900 − 850	C–C group	Stretch
720–820	C–H group	Rocking

Like Chopra et al.^3^, new OH group peaks appeared at 3750 cm^− 1^, corresponding to free hydroxyl (-OH) groups possibly due to hydrolytic degradation under UV + RH exposure. The weak band at 3750 cm^− 1^ suggests free non-hydrogen bonded hydroxyl groups at low concentrations often appearing at surfaces, indicative of chain scission and surface oxidation of PLA^28^. Splitting of the CH and CO bonds was observed at 1370 cm^− 1^ and 1080 cm^− 1^, respectively, and the C=C stretch was shown owing to sp^2^ CH. As biopolymers predominantly degrade by Norrish Type I, Norrish Type II, or both reactions when exposed to UV rays, the loss of peak intensity in the C-O stretch (1250–1050 cm^− 1^) and the characteristic C=O δ-lactone (1750 cm^− 1^) are indicative of the α-scission reactions (Norrish Type I)^16^. The Norrish Type II reactions that present as an abstraction of hydrogen are visible as the loss of intensity of CH groups at 760 cm^− 1^, 1450–1350 cm^− 1^, and the shift in peaks observed from 2925 to 2850 cm^− 1^ (sp^3^ to sp^2^ hybridisation)^3^. Furthermore, the presence of C=C group (~ 1630 cm^− 1^)^29^ occurring subsequently due to prolonged UV + RH exposure of over 1200 h, led to β-scission of the alkyl radical that breaks the C-C bonds [900–850 cm^− 1^], forming C=C groups. These structural changes can significantly compromise the mechanical properties of the FDM-PLA, leading to decreased tensile strength and embrittlement.

As ATR-FTIR is inherently surface-sensitive, the chemical changes reported here primarily reflect surface degradation processes. These surface-level modifications are considered contributory to tensile failure through embrittlement and crack initiation, rather than being direct measures of bulk chemical degradation. It should be noted that the accelerated UV-B exposure employed in this study represents intensified and spectrally constrained conditions relative to natural sunlight. Consequently, the observed degradation pathways, including signatures consistent with β-chain scission, reflect accelerated mechanistic trends rather than a direct one-to-one representation of natural outdoor degradation.

### Tensile performance

**Fig. 3:**
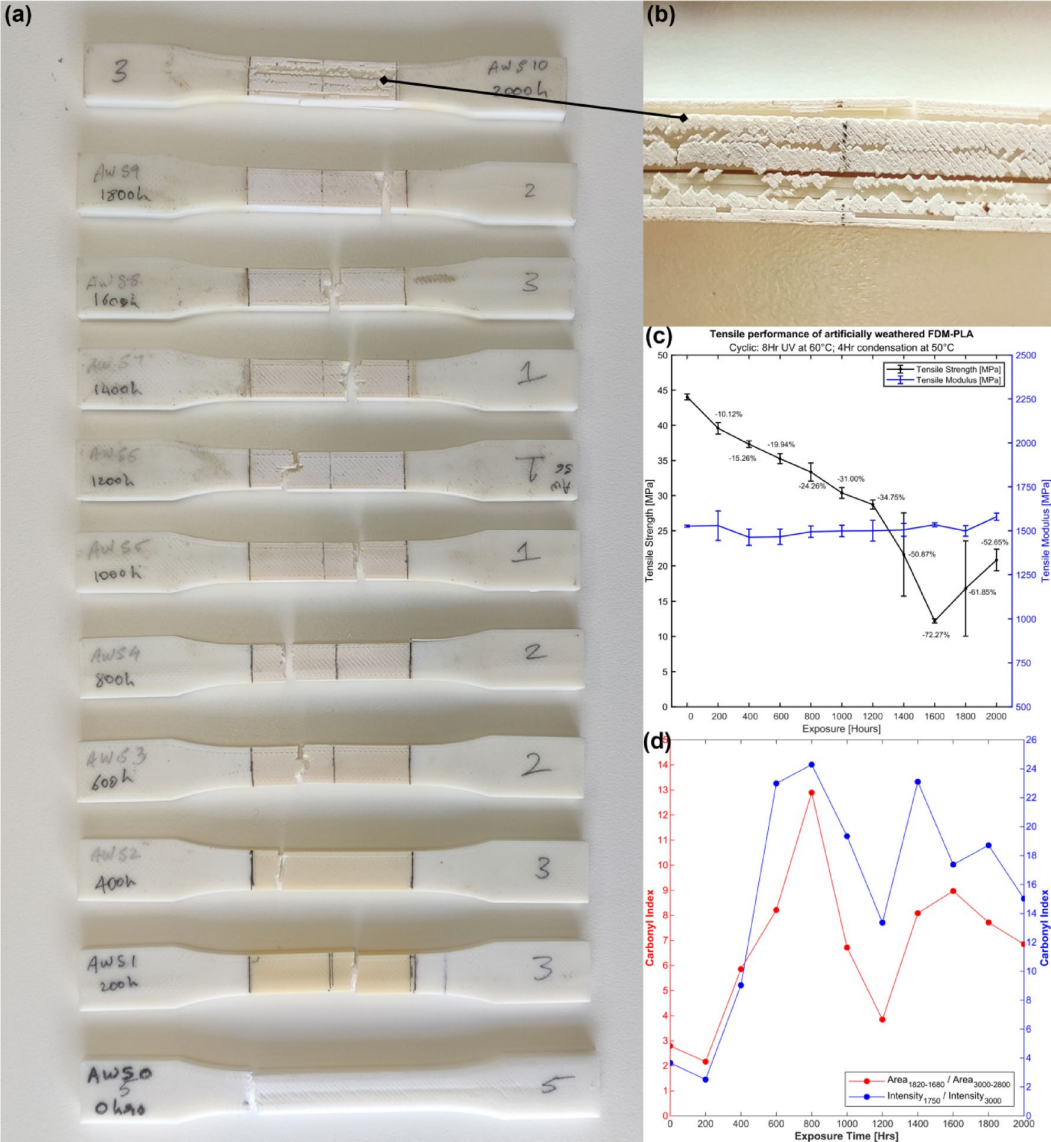
(**a**) Temporal changes in FDM-PLA tensile test coupons exposed to UV + RH accelerated weathering for up to 2000 h. (**b**) Surface cracks were observed on the gauge length of the 2000-hour sample. (**c**) Degradation of the tensile performance in the weathered test coupons over prolonged exposure. (**d**) Change in the test coupons’ carbonyl index (CI) over prolonged UV + RH exposure.

Three MEX-PLA tensile coupons were tested for every 200 h of UV + RH exposure at 60 °C and 50 °C, respectively, up to 2000 h of exposure. Figure 3a illustrates the progressive change in colour and surface roughness of the specimens over prolonged periods of UV exposure, leading to cracks and flaking of the topmost layer (Fig. 3b). A significant decline in tensile strength (Fig. 3c) was observed with UV + RH exposure, starting at a 10% loss at 200 h, followed by a near 5% decrease with every consecutive 200-hour exposure. After 1200 h, the tensile strength exhibited large deviations, likely due to increased brittleness. This behaviour is consistent with prior observations that the tensile response of FDM-based PLA is strongly governed by defect states such as voids, cracks, and interfacial discontinuities, and that modification or collapse of these features through post-processing or hybrid manufacturing routes can lead to substantial changes in tensile performance, independent of polymer chemistry^30^.The increased brittleness correlates with the observed slowdown or plateau in the rate of carbonyl group formation after a sharp increase up to 800 h (Fig. 3d). The subsequent fluctuation in the carbonyl index after 1200 h may indicate saturation of surface oxidation, the emergence of competing degradation mechanisms, and/or partial loss of degraded surface material due to cracking and flaking under prolonged exposure. Interestingly, the tensile modulus remains unaffected by the prolonged exposure and may be attributed to the increasing crystallinity of the samples, which has been shown to increase the tensile modulus of the weathered samples^18^.

While three specimens were tested per exposure interval to establish repeatability, the observed scatter at prolonged exposure durations is interpreted in a trend-based context. Increased variability beyond 1200 h is attributed to embrittlement and damage sensitivity, and larger sample sizes would be required for rigorous statistical confidence.

### X-ray diffraction (XRD)

**Fig. 4:**
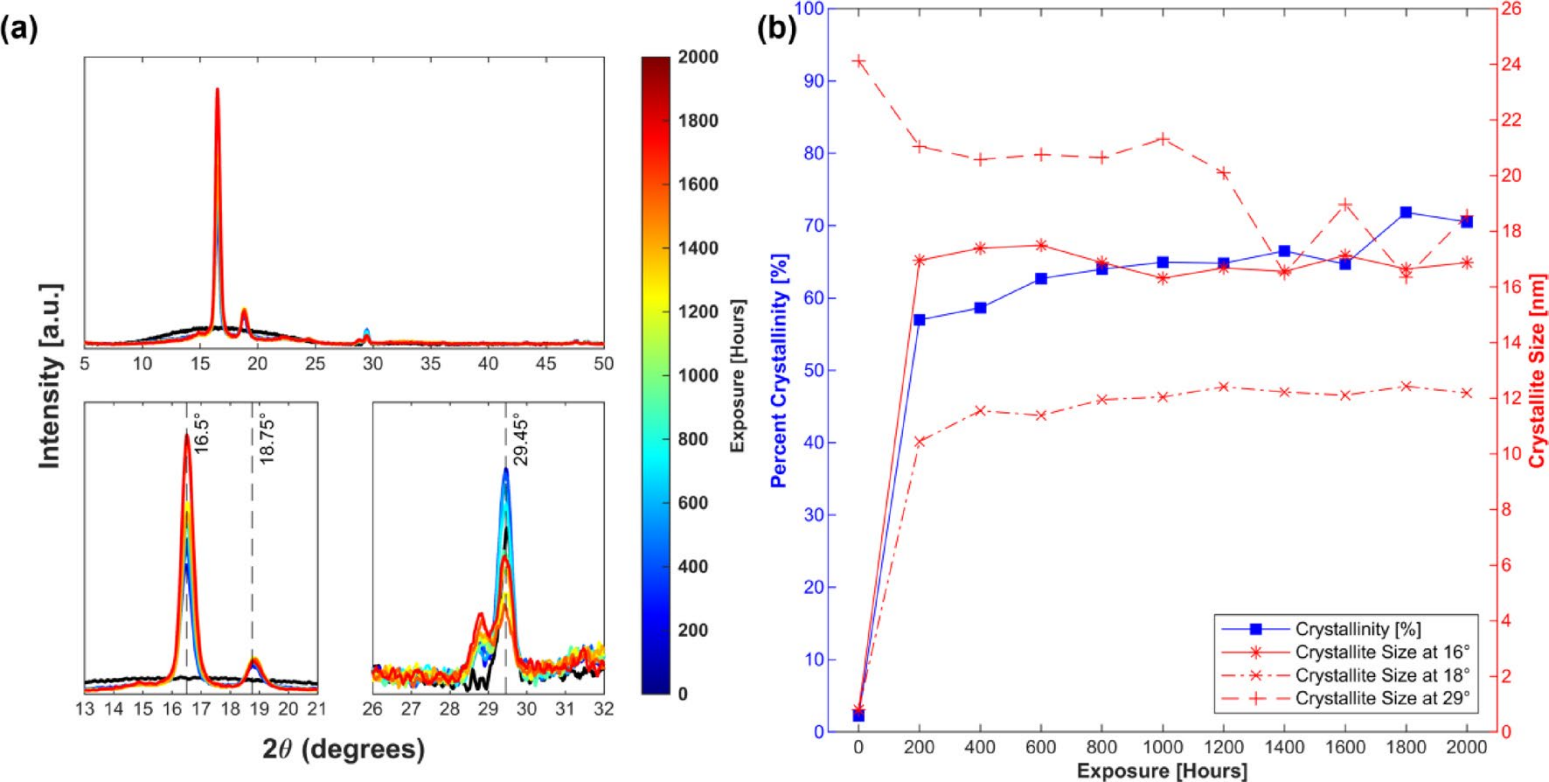
(**a**) Temporal change in XRD spectra with identified peaks observed at 2θ = 16.5°,18.75° and 29.45°, respectively, for progressive weathering exposure and (**b**) Change in per cent crystallinity (blue) and crystallite size (red) at each identified peak for 0–2000 h of accelerated UV + RH weathering.

XRD patterns were scanned in the gauge length of the exposed samples to evaluate the effect of prolonged UV + RH exposure on the crystallisation of semi-crystalline FDM-PLA. Figure 4a illustrates the semi-crystalline PLA with a broad amorphous halo and crystalline peak centred around 2θ = 16.5° and 29.5°, respectively, indicative of a predominantly amorphous structure. Upon UV + RH exposure, the amorphous halo resolved into two distinct characteristic α-phase crystalline peaks at 2θ = 16.5° and 18.75° as a result of crystallisation^19^, with increasing intensity for the former while the intensity remains unchanged for the latter. Interestingly, the crystalline peak at 29.45° decreases over continued exposure and is ascribed to the β-crystals, however, overlapping reflections or degradation-induced structural rearrangements cannot be entirely ruled out under the present experimental conditions^31,32^.

Figure 4b summarises the crystallinity (%) and crystallite size for the three distinct peaks observed at 2θ = 16.5° (200/110),18.75° (203) and 29.5° (0010), respectively. The near amorphous PLA with 2.24% crystallinity crystallises to 57% with 200 h of UV + RH exposure and steadily increases up to 71.84% with prolonged exposure up to 2000 h. Sawpan et al. also noted that the degree of crystallinity for artificially weathered PLA could exceed 50%^3,19^. While the crystallite size for 16.5° and 18.75° peaks shares a similar trend, the peak at 29.5° decreases with exposure times from 24.12 nm to 16.35 nm. These findings suggest that the UV + RH weathering induces crystallisation in the FDM-PLA samples, leading to a more crystalline and potentially less stable material, which translates to a decline in the tensile performance of these specimens.

Owing to the open chain ends created by the chain scission reaction, the content of amorphous segments in the polymer structure increases^33^. As the broken segments have higher freedom to form more organised structures, they try to crosslink to stabilise the structure, ultimately increasing crystallinity. Although this may increase the modulus^3^, the chain scission is more detrimental than any further cross-linking. However, the exact cause for this behaviour can be complex. DSC was employed to analyse the changes in the polymer’s thermal properties to understand the specific changes in the weathered PLA fully.

### Differential scanning calorimetry (DSC)

**Fig. 5:**
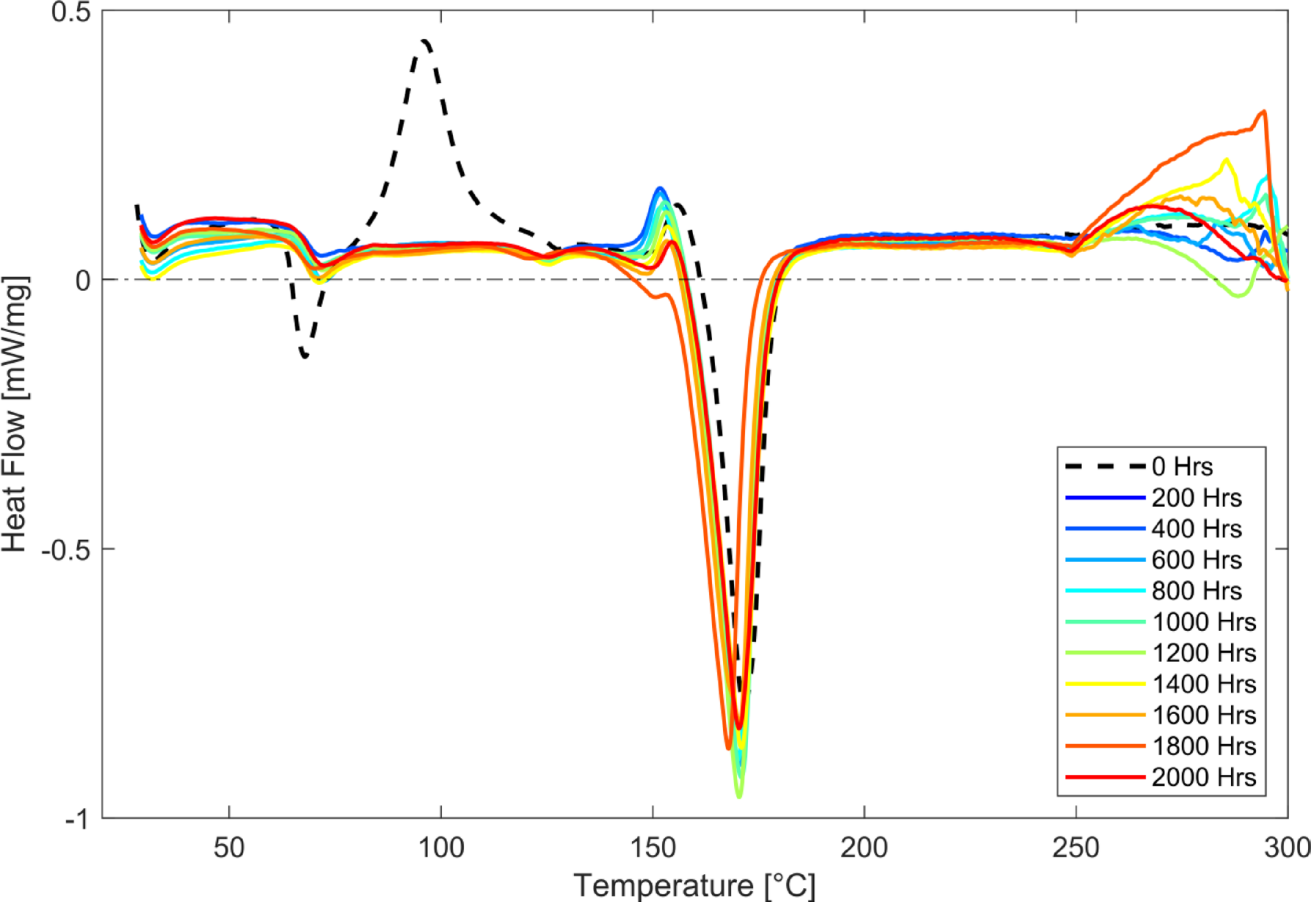
Change in heat flow from differential scanning calorimetry (DSC) of artificially weathered FDM-PLA over prolonged UV + RH exposure.

FDM-PLA tensile coupons were shaved at the exposed gauge length section and analysed using DSC to understand the changes in thermal behaviour over time of weathering exposure. The DSC curves were baseline corrected and overlayed in Fig. 5. The cold crystallisation peak (T_c_) at 96 °C is only visible in the unexposed (0 h) samples, indicative of the semi-crystalline nature of the polymer. The T_c_ disappears when exposed to UV + RH weathering, confirming the reduced amorphous phase, likely due to the chain scission degradation reaction, increasing the weathered FDM-PLA’s crystallinity. The increased crystallinity is confirmed by the XRD patterns obtained for these samples.

The increase in crystallinity observed by XRD and the disappearance of the cold crystallisation peak in DSC indicate progressive ordering of PLA chains during UV + RH exposure. While increased crystallinity contributes to stiffness retention, the concurrent chain scission and embrittlement dominate tensile strength degradation. This competing effect explains the observed divergence between relatively stable tensile modulus and continuously decreasing tensile strength.

The glass transition temperature (T_g_) at 67 °C shifts higher to 72 °C and nearly loses the area under the peak. The smaller peak is a common consequence of the crystallisation and Norrish chain scission reactions during weathering. The increased crystallinity and cross-linking limit chain mobility, making it difficult for the polymer to transition from a glassy to a rubbery state. The shift in T_g_ is attributed to a combination of annealing of the amorphous PLA and hydrolytic degradation^19,34^. The melting peak (T_m_) initially gets sharper with UV + RH degradation, indicative of increased crystallinity; however, prolonged exposure causes the T_m_ to shift lower from 171 °C to 168 °C for 1800 h. The combined effect may suggest a complex mechanism between partial degradation and the impact of any plasticisers. Additionally, the shift in T_g_ accompanied by the subtle exothermic peaks before thermal events indicate higher residual stresses in the exposed samples that may interfere with the T_m_ reading. A second heating is recommended to release stored energy and further understand the change in the thermal behaviour of the polymer samples.

It is emphasised that the crystallinity evolution observed in this study arises from coupled thermal, hydrolytic, and photochemical effects inherent to accelerated weathering conditions. These contributions cannot be uniquely decoupled within the present experimental framework.

## Conclusion

This study establishes the time-dependent effects of accelerated weathering of MEX-AM PLA. It was noted that prolonged exposure leads to β-scission of the alkyl radical that breaks the C-C bonds, forming C = C groups. Further, the FDM-PLA specimens indicate over 50% crystallisation within 200 h of cyclic UV + RH, continuing up to 72% at 2000 h of exposure. These structural changes can significantly compromise the mechanical properties of the FDM-PLA, leading to decreased tensile strength and embrittlement. A significant decline in tensile strength was observed with UV + RH exposure, starting at a 10% loss at 200 h, followed by a nearly 5% decrease with every 200-hour exposure. The study aimed to establish the time-dependent trend between weathering, mechanical properties, degradation time, crystallinity, and thermal properties. However, these tests were carried out in a simulated environment, and it is not straightforward to correlate these results with natural outdoor conditions.

It should be noted that accelerated weathering provides controlled and intensified UV and humidity exposure to rapidly investigate degradation mechanisms under repeatable conditions. In contrast, natural outdoor exposure involves fluctuating variables such as diurnal temperature changes, variable UV spectra, seasonal humidity, and environmental contaminants. Consequently, accelerated weathering reveals mechanistic trends but does not directly translate to service-life predictions, which require long-term outdoor validation. Hence, these results may not determine MEX-AM PLA’s durability in different environments. Future studies will explore stabilization strategies such as UV absorbers, antioxidants, coatings, polymer blending, and composite formulations to mitigate weathering-induced degradation in MEX-AM PLA. Given the known sensitivity of MEX-PLA mechanical response to process-induced features such as void architecture and raster configuration, quantitative degradation trends are expected to vary across different print parameter sets^35^.

## Data Availability

The authors declare that the data supporting the findings of this study are available within the article. The generated datasets are also available from the first and the corresponding authors on reasonable request.
